# Design of a Highly Sensitive Reduced Graphene Oxide/Graphene Oxide@Cellulose Acetate/Thermoplastic Polyurethane Flexible Sensor

**DOI:** 10.3390/s22093281

**Published:** 2022-04-25

**Authors:** Yujie Yang, Tan Yi, Yang Liu, Hui Zhao, Chen Liang

**Affiliations:** 1 College of Light Industry and Food Engineering, Guangxi University, Nanning 530004, China; yangyujie0985@163.com (Y.Y.); yitansysu@163.com (T.Y.); zhh@gxu.edu.cn (H.Z.); liangchen@gxu.edu.cn (C.L.); 2 Guangxi Key Laboratory of Clean Pulp & Papermaking and Pollution Control, Guangxi University, Nanning 530004, China; 3 Guangxi Bossco Environmental Protection Technology Co., Ltd., Nanning 530000, China

**Keywords:** electrospinning, porous fiber, flexible strain sensor, high sensitivity

## Abstract

As a substitute for rigid sensors, flexible sensing materials have been greatly developed in recent years, but maintaining the stability of conductive fillers and the stability of micro-strain sensing is still a major challenge. In this experiment, we innovatively prepared a polyurethane-based cellulose acetate composite membrane (CA/TPU) with abundant mesopores through electrospinning. Then, we reduced graphene oxide (rGO)—as a conductive filler—and graphene oxide (GO)—as an insulating layer—which were successively and firmly anchored on the CA/TPU nanofiber membrane with the ultrasonic impregnation method, to obtain an rGO/GO@CA/TPU sensor with a GF of 3.006 under a very small strain of 0.5%. The flexibility of the film and its high sensitivity under extremely low strains enables the detection of subtle human motions (such as finger bending, joint motion, etc.), making it suitable for potential application in wearable electronic devices.

## 1. Introduction

In recent years, with the rapid development of fields within electronic technology, such as intelligent robots, flexible wearable devices, mobile intelligence, and electronic skin, the research and development of functional flexible sensors has attracted increasing levels of attention [1,2,3,4]. Due to the narrow strain range and easy plastic deformation of rigid sensors, such as traditional metal foil and semiconductor strain sensors, which have poor stretchability (ε < 5%) [5,6], it is increasingly difficult to meet the requirements of new conductive materials for a high strain-sensing ranges and deformable sensing. More importantly, the flexible sensor manufacturing process is simple, and such sensors have a low cost and light weight. Flexible sensors can respond to external signals in real time and in any form, and they can provide output in the form of electrical signals, which also makes them better for applications as wearable devices, in device motion detection, as health testing equipment, amongst other fields [1,2,4]. The response of a flexible sensor is realized by the conductive unit, which forms a path, and regular changes in the conductive unit due to external stimuli [7]. Except for a few structural self-conductive polymers, most flexible sensors are realized primarily with composite conductive polymers that are composed of non-conductive polymer materials mixed with conductive substances [8]. The electron transport mechanism of this type of composite conductive polymer is mainly explained by three theories: the “conducting path”, “tunneling effect” and “field electron emission” [9,10,11].

At present, there are many kinds of materials used for flexible sensors, such as polydimethylsiloxane (PDMS) [12], thermoplastic polyurethane (TPU) [13,14], polyvinylidene fluoride (PVDF) [15], and so on. Among them, fiber materials have attracted extensive attention due to their advantages of having a high specific surface area, being lightweight, having a simple preparation process, and low cost. As is well known, with the rapid development of the theory of the electrospinning process and nanocomposite materials, electrospinning has become the most versatile and feasible technical means of producing continuous one-dimensional ultrafine fibers [16,17]. In addition, the morphology of the fibers can be adjusted during the electrospinning process to obtain different structures to meet different needs. The use of electrospinning to fabricate sensors has also received much attention. For conductive fillers, metal/metal oxide nanoparticles [18], carbon nanotubes (CNTs) [13,15], metal nanowires [19], reduced graphene oxide (rGO) [14], etc., are commonly used. For the composite method of using conductive fillers and flexible substrates, the most common methods are blending before film formation and dipping after film formation. For example, Li and their co-workers fabricated a TPU-based flexible piezoresistive pressure sensor in an electrospun fiber network (TPUN), which was successfully decorated with c-MWCNTs and impregnated in a TPUN with excellent electrical conductivity [20]. Tang et al. prepared a CNT/TPU composite nanofiber yarn with elongation at break as high as 476% by uniformly dispersing CNT into TPU and blending to obtain a spinning solution and by using a multi-needle liquid bath electrospinning method. The nanofibers were then coated with CNTs by dip coating, resulting in a strain sensor that exhibited a high relative resistance change (440%) at 140% strain [13]. In order to maintain the stability of the sensor’s sensing, that is, to make it more less easy for the conductive filler to fall off of the flexible substrate, we innovatively constructed a microstructure on the fiber that can firmly anchor the conductive filler to improve the sensing effect of the sensor.

For strain sensors, a suitable strain range and a certain sensitivity are two important factors to be considered in the design [21,22]. Cao et al. designed a silver nanowire/polyurethane (AgNW/PU) composite fiber with a shell-core structure, and by adjusting the adhesion layer between the silver nanowire and the PU substrate, they prepared fiber-based strain sensors with different GFs and working ranges. Among them, the AgNW/PU-9.2 wt%/5 min fibers had a wide working range of 0–50% and a large GF of 940; however, the GF was still in the low strain range (at <10%, the GF was almost 0) [23]. In addition, Wang et al. fabricated a sensor that exhibited a high sensitivity under extremely large tensile strains (a gauge factor of 8962.7 at 155% strain). They fabricated the sensor in a conductive polymer embedded with carbon black (CB) particles in an electrospun TPU fiber membrane matrix with a tunable scaffold network. This work demonstrates the effects of three-dimensional scaffold network structures, constructed at different rotational speeds of the collection device on the electrical response of the a TPU/CB strain sensor during electrospinning [22]. Although sensors with ultra-high sensitivity under extremely large strains have been extensively studied, sensors that can record extremely small strains also have great implications for medicine and human health.

**Figure 1:**
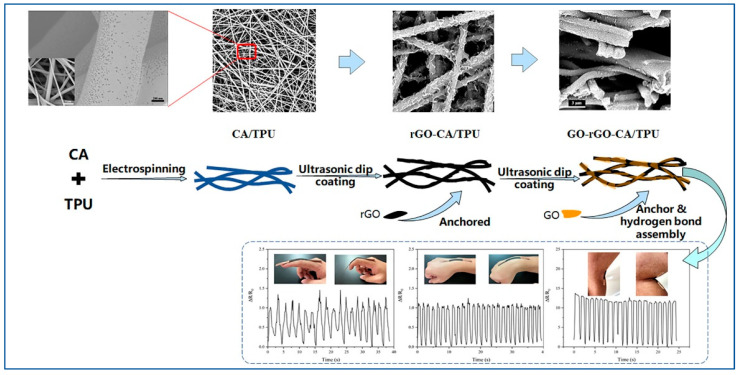
Schematic diagram of the whole preparation process of the GO/rGO@CA/TPU nanofiber film.

Therefore, this paper innovatively designs a sensor with high sensitivity under an extremely small strain. Thermoplastic polyurethane (TPU), whose toughness can reach 390.2 MJ·m^−3^ [14,24] and cellulose acetate (CA) which has good air permeability and natural polymer material [25,26], were chosen to comprise the substrate of the sensor. For the conductive filler, reduced graphene oxide (rGO) was chosen. This is because of the two-dimensional structure of graphene, which can form electron transport paths through the contact between graphene sheets. A small change in the overlapping area or the relative position between graphene layers can cause a huge change in the conductive structure of the film, which is manifested as a change in the material resistance or capacitance. The easily altered conductive structure also makes graphene a promising candidate for conductive sensing [27,28]. Then, through the difference in the boiling points of the mixed solvents and the difference in the solubilities of each solvent with respect to the raw material during the electrospinning process, the mesoporous nanofiber film was prepared in one step during the electrospinning process, which made the circuit structure of the sensor more stable, and it improved the sensitivity of the sensor. Finally, the nearly insulating graphene oxide (GO) was wrapped with the conductive filler on the surface of the nanofibers through hydrogen bonding under the action of ultrasonic waves, and a GO/rGO@CA/TPU sensor with a stable circuit structure was obtained. The experimental process is shown in Figure 1. This research has contributed new ideas for the design of the circuit structure of flexible sensing materials and for the improvement of sensitivity.

## 2. Materials and Methods

### 2.1. Materials

CA powders were purchased from Macklin (Mw = 60,000, AR, Shanghai, China) and TPU granules were purchased from Bayer (90A, Germany); these were used to prepare the solutions for electrospinning. The rGO (>99%, Shanghai Aladdin Biochemical Technology Co., Ltd., Shanghai, China) and GO (99.7%, Shanghai Aladdin Biochemical Technology Co., Ltd., Shanghai, China) were purchased from Nanjing Xianfeng Nano Co., Ltd. (Nanjing, China). *N*,*N*-dimethylacetamide (DMAc, AR, Tianjin Zhiyuan Chemical Reagent Co., Ltd., Tianjin, China), acetone (PA, AR), and absolute ethanol were of analytical grade, and were used without further purification.

### 2.2. Preparation of the Flexible Sensor

#### 2.2.1. Preparation of the CA/TPU Composite Film

We prepared the CA/TPU composite film through electrospinning. First, we configured the spinning solution. In this experiment, CA and TPU were used as solutes, and DMAc and PA were used as solvents. Spinning solutions with different mass fractions of 12–20% were prepared by stirring under a magnetic stirrer for 2 h. Second, electrospun fibers were prepared using a syringe loaded with a 23 G metal needle. The feeding speed of the electrospinning machine was fixed at 0.18 mm/min, the distance between the needle and the receiver was fixed at 15 cm, and the voltage used for spinning was 12.5 kV. All experiments were performed at room temperature (T = (27 ± 2) °C, RH = (50 ± 2)%). 

#### 2.2.2. Preparation of the GO/rGO@CA/TPU Film

The anchoring of graphene was the key to the conductivity of the composite film. First, 1.5 mg/mL rGO ethanol dispersion and a 0.5 mg/mL GO ethanol dispersion were prepared. Then, the CA/TPU nanofiber spinning films were immersed in the prepared rGO and GO dispersions and sonicated for 20 min. After completion, the sample strips were taken out, rinsed 3 times with water and ethanol, and then vacuum-dried for 6 h to finally obtain the GO/rGO@CA/TPU film that we needed.

### 2.3. Characterization

The surface morphology of the nanofibers was observed with a desktop scanning electron microscope (SEM, Phenom ProX type Phenom, Eindhoven, The Netherlands, acceleration voltage: 10 kV) and a field–emission electron microscope (FEM, Sigma 300 type, Zeiss, Oberkochen, Germany, acceleration voltage: 5 kV). An automatic specific surface area tester (BET, TriStarII;3020, Micromeritics Instrument Ltd., Norcross, GA, USA) was used to measure the specific surface area and pore size of the spinning film. The degassing temperature of the fiber film was 110 ℃, and the degassing time was 6 h. The surface element and chemical functional group changes of the sample were characterized with an X-ray photoelectron spectrometer (XPS, ESCALAB 250XI+ X, Thermo Fisher Scientific, Waltham, MA, USA) with a spot diameter of 500 μm. The film strain response test was performed with an electrochemical workstation (AUTOLAB PGSTAT302N, Metrohm, Herisau, Switzerland), and the sampling time was 0.1 s. The viscosity of the spinning solution is measured with a micro mixing rheometer (Haake Minilab, Thermo Fisher Scientific, Karlsruhe, Germany), the measurement mode was the frequency sweep mode (25 ℃, shear rate 0~300 S^−1^), and the measurement was the zero–time shear viscosity. 

### 2.4. Sensor Sensitivity Measurement

The sensitivity of the sensor is expressed by the gauge factor (GF), which represents the ratio of the relative value of the change in the sensor’s resistance to the applied strain, and the formula for its calculation is given in Equation (1) [29]:

(1) $$\mathrm{GF}=\frac{\Delta R/R_{0}}{\varepsilon }$$

where GF is the strain sensitivity coefficient, R_0_ represents the resistance value when no strain is applied, ΔR is the difference between R_0_ and the resistance value at any time during the stretching process, and ε is the strain.

## 3. Results

### 3.1. Electrospinning of the Mixed Solution

**Figure 2:**
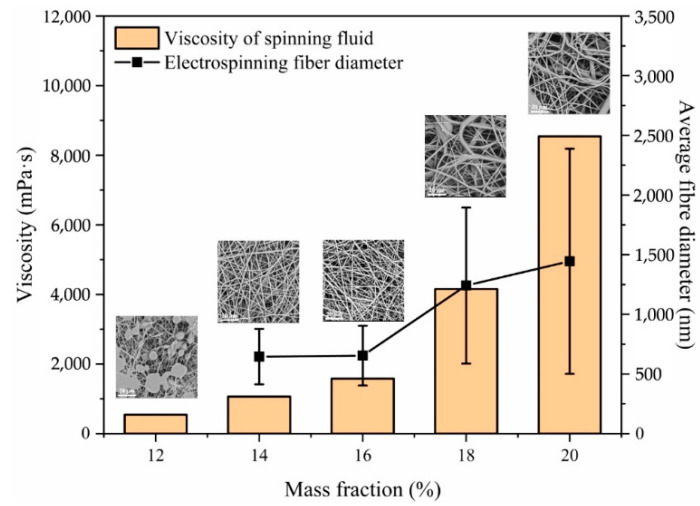
Effect of the viscosity of the spinning fluid on fiber morphology and the distribution of the fiber diameter.

Voltage is an important influencing factor in the electrospinning process. As the internal driving force of electrospinning, only when the applied electric field voltage is higher than the threshold voltage will the polymer solution be drawn to form Taylor cones and cracked and drawn to form jets [30,31]. In addition to the voltage, the viscosity of the polymer is also a key factor in the spinning process. The viscosity of the polymer solution or melt, the concentration, and the molecular weight are related to each other. Increasing the concentration or molecular weight can increase the viscosity of the solution. In the electrospinning process, only when the solution concentration reaches a certain critical value can the solution be stably split in the electric field and form a jet; with a change in the solution concentration from low to high, the fiber also shows the change in the bead, from spindle, to fine fiber, to thick fiber. Finally, when the concentration increases to a certain level, the electric field can no longer overcome the viscous resistance of the solution, and the solution will also form larger droplets at the tip of the needle, blocking the needle [32,33]. We experimented with spinning solution concentrations ranging from 12% to 20%, and Figure 2 shows the effect of the spinning solution concentration on the fiber morphology and the distribution of the fiber diameter. The experimental results show that when the concentration was 12%, the electrospray could not effectively stretch and dry to form fibers. Instead, a large number of irregular beads appeared on the surface and inside of the deposited fibrous film in the form of liquid beads. When the solution concentration reached 14%, the solution was able to form a stable jet in the electric field. It can be seen from the SEM image that uniform and stable fibers could be formed when the solution concentration was 14% and 16%; however, when the concentration reaches more than 18%, the diameter of the fiber becomes larger and uneven. This is because when the concentration is too high, the molecular chains in the polymer solution are too tightly entangled, so the electrospray cannot be effectively stretched during the spinning process, and cleavage occurs during the formation of uniform and stable fibers; therefore, for this experiment, we chose a concentration range of 14–16%.

### 3.2. Analysis of the Pore Size and Morphology of the Electrospun Fibers

The main principle used in this experiment to prepare porous materials was nonsolvent-induced phase separation (NIPS) [34,35], by using the difference between the boiling points of the spinning solution solvents and the differences in the solubility of the solvents with respect to those of different raw materials. The boiling point of acetone under standard conditions is 56.53 °C, and that of DMAc is 164–166 °C. The huge difference in boiling points makes acetone volatilize first in the spinning process, forming air pockets in the jet, which has not yet been volatilized and solidified, and diffusing to form pores in the uncured jet. At the same time, in this process, the solubility of acetone for CA is much higher than that of TPU and DMAc has good solubility for CA and TPU; therefore, during the rapid volatilization of acetone, the pores constructed by the acetone can be quickly partially cured by the precipitated TPU, and will not be filled by the flowing solution. This further ensures the uniformity and stability of the pores on the fiber surface, and holes with different pore sizes can be constructed on the fiber surface according to the difference in the fluidity of the spinning solution [36,37]. 

**Figure 3:**
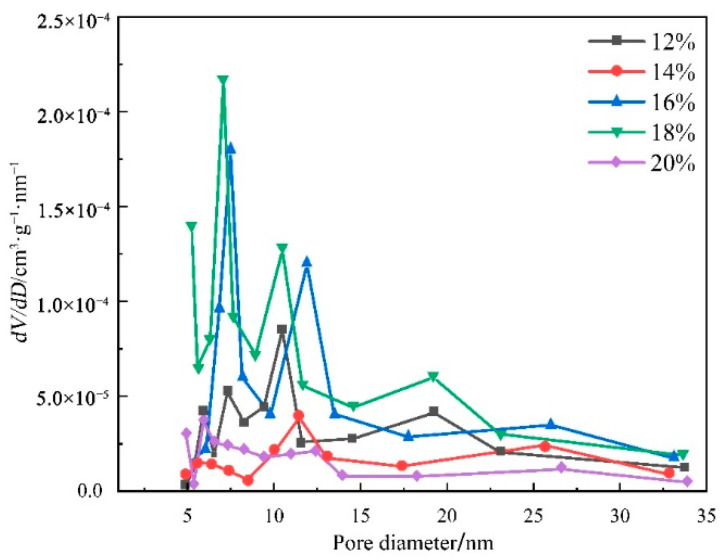
BET analysis of the electrospun fiber surface at different concentrations.

Figure 3 shows a BET analysis diagram of the fiber surface with different spinning solution concentrations. Combined with the specific surface area and average pore size corresponding to different concentrations of spinning solutions in Table 1, it can be seen that, with the increase in concentration, the pore size showed a trend of first increasing and then decreasing, and the specific surface area also showed a trend of first increasing and then decreasing, which was consistent with the change trend in pore size. The spinning solution had a high fluidity at a low concentration, and the air pockets formed by the volatilization of acetone were easily refilled by the spinning solution with a high fluidity, which caused the pore size to decrease. This was because as the concentration of the solution increases, its fluidity decreased and the pore size gradually increased; however, when the concentration increased to a certain level, the molecules in the spinning solution were tightly entangled, and the air pockets formed during the volatilization of acetone could not grow in the fiber, causing the pore size to decrease. The change in the specific surface area was caused by the change in the fiber pore size [38]. The higher specific surface area of the nanofiber film greatly improves the physical adsorption of rGO and GO, and the appropriate pore size on the nanofiber surface also causes rGO and GO to have more stable anchoring sites; therefore, our subsequent experiments will be carried out with spinning films with concentrations of 14% and 16%.

**Figure 4:**
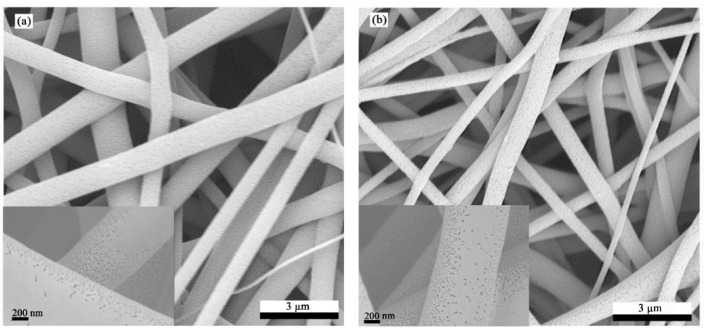
SEM micrographs of the electrospun fiber surface: (**a**) 14% concentration and (**b**) 16% concentration.

The concentration of the spinning solution did not appear to exceed 50 nm in the range of 12% to 20%, and the peak in 5 to 35 nm indicated that the surface of the fiber was rich in mesopores. It was found that when the fiber concentration was 12%, 18%, or 20%, spikes appeared at multiple locations and extended to the range of the micropores, the pore size distribution on the fiber surface was uneven, and there were micropores on the fiber surface. There were very few micropores and mesopores, and most of the pores on the fibers were larger than 50 nm [39]. For example, Li et al. used the same principle to electrospin polylactic acid/chitosan and found that pores of about 60 nm were generated on the fibers, and they applied this to air purification [40]. When the spinning solution concentration was 14% or 16%, the viscosity of the spinning solution was moderate, and the air pockets formed by the rapid volatilization of acetone could expand and grow. This is because the solubility of acetone for CA is much higher than that of TPU, and DMAc has good solubility for CA and TPU; therefore, the pores formed after acetone volatilization could be quickly precipitated. The solidified TPU was partially stable, ensuring the uniformity and stability of the pores [41]. Figure 4 presents an SEM image of the morphology and pore distribution of the mesopores on the fiber surface when the spinning concentration was 14% and 16%. The mesopores on the fiber surface were round or short and rod-shaped, and they were densely distributed on the fiber surface. The appropriate pore size on the fiber surface also provided a more stable anchor point for rGO and GO, which improved the stability of the two on the fiber surface and prevented the performance of the nanofiber film from being affected by the shedding of rGO.

### 3.3. Characterization of Composite Film

**Figure 5:**
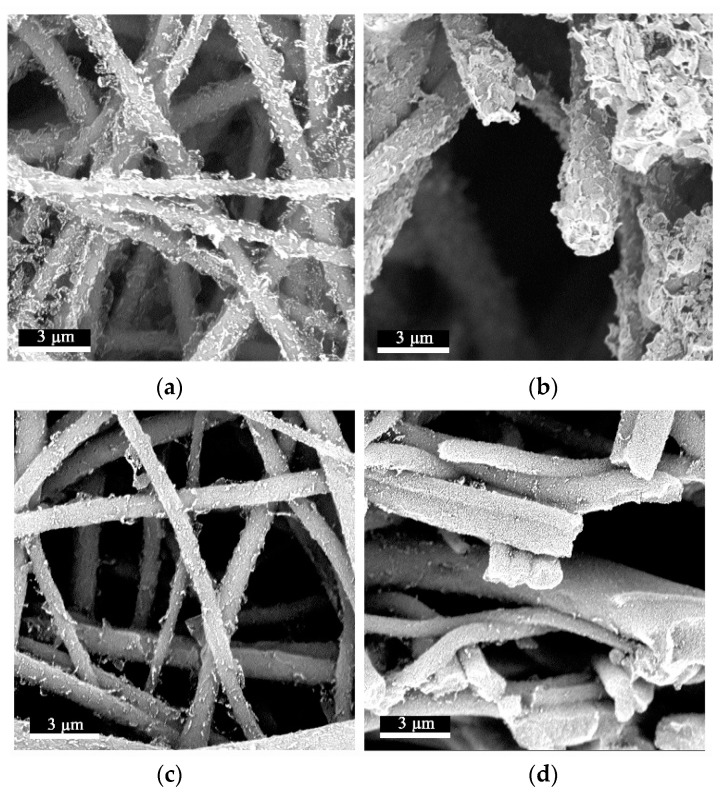
The surface micromorphology of anchored graphene fibers: (**a**) plan view of nanofibers after anchoring rGO; (**b**) cross-sectional view of nanofibers after anchoring rGO; (**c**) plan view of nanofibers after anchoring GO; (**d**) anchor cross-section of nanofibers after GO.

We chose rGO as the nano-conductive filler, used the CA/TPU electrospun composite film as the substrate, and selected the insulating GO to prepare the flexible sensor. In order to observe the surface and cross-sectional micromorphologies of the CA/TPU nanofiber films after anchoring graphene, SEM was used to observe the surface and cross-sectional micromorphologies of the films. As shown in Figure 5a,b, the flaky rGO sheets formed a scaly structure on the surface of the nanofibers [42], and the overlapping between fibers also caused the film to form a stable three-dimensional conductive network. In addition, it can be seen in Figure 5c,d that, after further ultrasonic anchoring of GO, the surface of the nanofibers became smooth and flat, and there were not many lifted rGO sheets on the surface of the fiber. This phenomenon was mainly due to the fact that the oxygen-containing groups on the surface of rGO were reduced, and the lamellae could not form hydrogen bonds with the nanofibers after anchoring in the micropores on the fiber surface. In addition, because the surface groups of rGO were removed, the dispersibility of rGO in ethanol was poor, and the rGO sheet could not be fully spread in the dispersion, which also caused the lifting of the rGO layer after anchoring [43]. After the secondary anchoring of GO, and since the surface edge of GO had a large number of oxygen-containing groups (–OH, –COOH, –CH(O)CH–, etc.), it was very easy for these groups to interact with the hydroxyl and carboxyl groups on the fiber surface, so GO was firmly adsorbed on the fiber surface. At the same time, due to the electrostatic repulsion of the surface groups of GO, GO had better dispersibility during ultrasonication and can be repeatedly spread in the ethanol solution. The fully stretched GO was also wrapped on the outer surface of the fiber under the action of ultrasound, and the warped rGO was wrapped inside, which also made the fiber surface smoother after anchoring GO [44,45].

**Figure 6:**
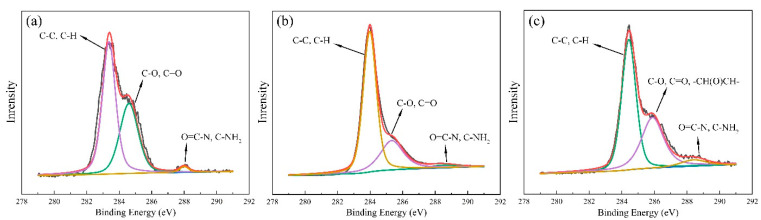
C_1S_ high-precision photon energy spectrum of the film: (**a**) CA/TPU film, (**b**) rGO@CA/TPU film, and (**c**) GO/rGO@CA/TPU film.

In order to explore the elemental content of the film and the change in the oxygen content of the film before and after anchoring GO, an X-ray photon spectrometer was used. As shown in Figure 6a, the binding energy at 283.4 eV was the characteristic peak of C–C and C–H, and the binding energy at 284.3 eV was the characteristic peak of the cellulose acetate pyran ring and the C–O and C=O bond in the structural unit; the binding energy of 288.05 eV was the characteristic peak of O=C–N in TPU [46,47]. Similarly to Figure 5a, the photon energy spectra in Figure 6b,c also show three main characteristic peaks. It can be seen in Table 2 that, after anchoring rGO, the carbon content in the film increased from the initial 78.40% to 89.86%. After anchoring GO, due to the large number of oxygen-containing groups carried on the GO surface, the carbon content of the film decreased from 89.86% to 78.34%, and the oxygen content increased from 8.39% to 18.32%. The decrease and then increase of oxygen content, and the increase and then decrease of carbon content, also confirm the successful anchoring of rGO and GO; however, it can also be seen in Table 2 that the nitrogen content had an upward trend after anchoring GO, which may have been caused by the fact that GO and rGO did not completely wrap the fibers. In addition, the problem that the package is not tight enough needs to be solved in follow-up experiments.

### 3.4. Strain Response Analysis of the Sensor

**Figure 7:**
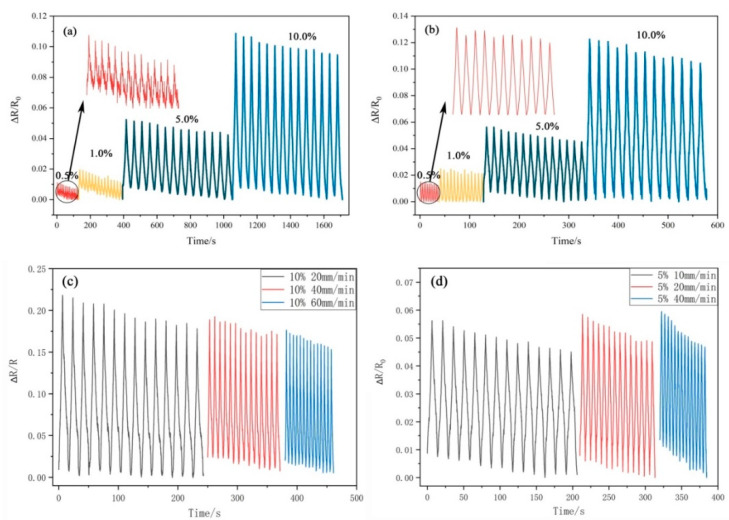
Tensile strain response of the CA/TPU film before and after graphene anchoring. (**a**) Resistance response curves of rGO@ CA/TPU under different strains; (**b**) resistance response curves of rGO/GO@CA/TPU under different strains; (**c**) resistance response curves of rGO/GO@CA/TPU at different stretching rates under 10% strain; (**d**) resistance response curves of rGO/GO@CA/TPU at different stretching rates under 5% strain.

Figure 7 presents a graph of the tensile strain response before and after graphene anchoring. Figure 7a,b are the ΔR/R_0_-t curves of the CA/TPU fiber film anchored with rGO and GO under different tensile strains, respectively. It can be seen that the curves all showed obvious regularities, and with the continuous increase in strain, the range of ΔR/R_0_ increased. Comparing (a) and (b) in Figure 7, although the rGO@CA/TPU sensing film could output a response signal with a mode variation as low as 0.5%, it can be clearly observed that the response signal of the rGO@CA/TPU film was unstable. Although peaks and troughs in the response signal could appear during the stretching process, the surface of the curve was rough and could not reflect the strain state of the film well. This was mainly because the thickness of the film continuously decreased during the small strain stretching process, and the distance between the nanofibers in the film kept decreasing, which caused the rGO layers raised on the surface of each fiber to contact each other, and the circuit structure between adjacent fibers was turned on. As a result, the structural stability of the circuit of the film was affected, and the curve oscillated within a single strain cycle. After the insulating GO was added, the rGO on the fiber surface was wrapped, and the reduction of the distance between the fibers after stretching did not cause the circuit structure between adjacent fibers to conduct, so the circuit structure of the film was more stable. In addition, the strain sensitivity factor (GF) of the sensor was improved after anchoring GO. As shown in Table 2, after anchoring GO, the GF of the flexible conductive films increased by 33.1%, 26.9%, 13.8%, and 27.6% at 0.5%, 1.0%, 5.0%, and 10% strain amounts, respectively. In particular, the sensitivity under extremely small tensile strains has been greatly improved.

Figure 7c,d show the response signal graphs of the flexible sensor at different strain rates for the 10% and 5% strains, respectively. It can be seen that, under different strain rates, the films could monitor the strain process and transmit it in real time; however, the sensing effect of the sensor was not good at a large stretching rate, which was mainly due to the insufficient elasticity of the nanofiber film due to the blending of CA, and the rebound rate was slow. Therefore, at a large stretching rate (such as 10%, 60 mm/min), the film could not return to its original state after the end of the previous stretching cycle and enter the next stretching cycle, which caused the relative positions of graphene on the fiber surface to be separated again before returning to the initial state [48]; therefore, the sheet resistance increased continuously during the cyclic stretching process, and the resistance response curve also showed a decreasing trend. At the same time, this was also the reason for why the GF of the GO/rGO@CA/TPU sensor decreased continuously with the increase in the strain variable during the testing process (Table 3); therefore, improving the rapid shrinkage ability of the film after stretching and reducing the continuous decrease of the response signal intensity with time have become urgent problems to be solved.

### 3.5. Human Motion Detection with the Flwxible Sensor

**Figure 8:**
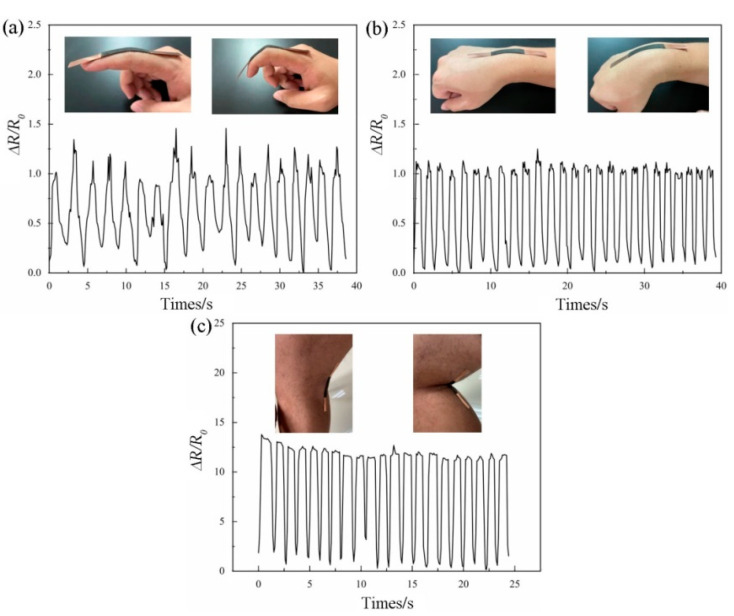
Monitoring of the motion of the human body with the functional flexible sensor. (**a**) Monitoring of the lateral flexion and extension of fingers, (**b**) monitoring of the lateral flexion of the wrist, (**c**) monitoring of the dorsal flexion and extension of the knee.

The flexible sensor is different from a traditional rigid sensor because of its flexibility and the advantages of adapting to large tensile strains, making it suitable for monitoring the motion of human joints; therefore, we applied it to some simple human motion monitoring to prove that it has possibilities for actual applications in the future [49,50]. As shown in Figure 8, the cellulose-based film was applied to the fingers, wrists, and knees of the human body, and the sensor was connected to the electrochemical workstation through the conductive glue on the copper foil to record the resistance response of the film during the movement of the corresponding position of the human body during a bending movement. Figure 8a presents a graph of the response when applying the film to the outside of the finger. Although the fingers flexed and stretched irregularly during this process, it was found that the film could still monitor the process of the charge in the finger state well without presenting a cluttered signal [21,23]. In Figure 8b, the film was attached to the outside of the wrist. With regular high-speed bending of the wrist, the film resistance response also showed regular vibrations; however, when the wrist returned to the initial position (the sensor was not deformed), due to small vibrations in the wrist, the sensor’s resistance response also had a small vibration at the peak position. Notably, as shown in Figure 8c, the film was attached to the inside of the knee. When we simulated the rapid flexion, flexion, and extension of the knee socket during running, the film exhibited an extremely high strain response with a ∆R/R_0_ of 13, which was also the strength of this sensor [14]. This indicates that the sensing sensitivity of the film under folded contact work was much higher than that under tensile strain. This also provides a new option for the application of cellulose-based sensing materials with poor resilience [51].

## 4. Conclusions

This article reports a method of using differences in the solvent boiling point and solubility to achieve phase separation, preparing a nanofiber material with rich mesopores on the surface by electrospinning in one step, and designing a sensor with a high sensitivity under an extremely small strain. The CA/TPU composite nanofiber films with uniform and stable nanofiber diameters and rich mesopores on the surface (pore size of about 10 nm) were obtained through electrospinning. Then, the ultrasonic dip coating–drying process was used to firmly anchor the rGO/GO on the fiber surface to obtain a stable and uniform flexible rGO/GO@CA/TPU sensor. The experimental results showed that rGO was uniformly wrapped on the surface of the CA/TPU fibers, and interconnected to form a good conductive network. The GO on the surface of rGO@CA/TPU prevented the overlapping of rGO during straining and provided a basis for obtaining stable electrical signals. The optimization of the circuit structure with GO also significantly improved the sensitivity of the sensor by 33.1% compared with that of rGO@CA/TPU. Finally, the sensor that we obtained was able to realize real-time monitoring of the strain process under an extremely low strain (0.5%), and its sensitivity could reach 3.006. In addition, the sensor is wearable and can detect subtle movements of the human body, including the bending of the fingers, bending of the elbows, and so on. It can be applied for medical detection and in sports health monitoring. This provides new ideas for designing flexible and high-sensitivity wearable electronic devices; however, during the experiment, we found that the anchoring and adsorption of the graphene nanosheets on the fiber surface were not tight. In addition, the tensile resilience of the sensor was poor, and the film only showed good test results under low strain tests. It is necessary to further improve the resilience performance of the cellulose-based electrospinning membrane after stretching in subsequent experiments to improve the versatility of strain sensing.

## Figures and Tables

**Table 1:** Specific surface area and average pore diameter of the composite nanofiber film.

Concentration	12%	14%	16%	18%	20%
Specific surface area (m^2^/g)	1.7754	4.5503	4.6869	4.2085	2.8931
Aperture (nm)	11.2880	8.1812	7.7864	6.8551	4.4871

**Table 2:** Changes in the elemental content of the nanofiber films during successive anchoring.

Electrospun Film	C_1S_ (%)	O_1S_ (%)	N_1S_ (%)
CA/TPU	78.40	15.56	6.04
rGO@CA/TPU	89.86	8.39	1.75
GO/rGO@CA/TPU	78.34	18.32	3.34

**Table 3:** The maximum strain sensitivity coefficient of each cellulose-based conductive film with different strain variables.

Strain/%	GF	
	rGO@CA/TPU	GO/rGO@CA/TPU
0.5	2.258	3.006
1.0	1.954	2.479
5.0	1.047	1.191
10.0	1.087	1.387

## Data Availability

Not applicable.
